# Absolute Depth Sensitivity in Cat Primary Visual Cortex under Natural Viewing Conditions

**DOI:** 10.3389/fnsys.2016.00066

**Published:** 2016-08-05

**Authors:** Ivan N. Pigarev, Ekaterina V. Levichkina

**Affiliations:** ^1^Institute for Information Transmission Problems (Kharkevich Institute), Russian Academy of SciencesMoscow, Russia; ^2^Department of Optometry and Vision Sciences, The University of Melbourne, ParkvilleVIC, Australia

**Keywords:** depth perception, primary visual cortex, V1, depth constancy, 3D, distance perception

## Abstract

Mechanisms of 3D perception, investigated in many laboratories, have defined depth either relative to the fixation plane or to other objects in the visual scene. It is obvious that for efficient perception of the 3D world, additional mechanisms of depth constancy could operate in the visual system to provide information about absolute distance. Neurons with properties reflecting some features of depth constancy have been described in the parietal and extrastriate occipital cortical areas. It has also been shown that, for some neurons in the visual area V1, responses to stimuli of constant angular size differ at close and remote distances. The present study was designed to investigate whether, in natural free gaze viewing conditions, neurons tuned to absolute depths can be found in the primary visual cortex (area V1). Single-unit extracellular activity was recorded from the visual cortex of waking cats sitting on a trolley in front of a large screen. The trolley was slowly approaching the visual scene, which consisted of stationary sinusoidal gratings of optimal orientation rear-projected over the whole surface of the screen. Each neuron was tested with two gratings, with spatial frequency of one grating being twice as high as that of the other. Assuming that a cell is tuned to a spatial frequency, its maximum response to the grating with a spatial frequency twice as high should be shifted to a distance half way closer to the screen in order to attain the same size of retinal projection. For hypothetical neurons selective to absolute depth, location of the maximum response should remain at the same distance irrespective of the type of stimulus. It was found that about 20% of neurons in our experimental paradigm demonstrated sensitivity to particular distances independently of the spatial frequencies of the gratings. We interpret these findings as an indication of the use of absolute depth information in the primary visual cortex.

## Introduction

It is generally recognized that reconstruction of 3D information from 2D retinal representations is one of the most important functions of the visual system. The most obvious mechanism of this function could be one based on the processing of binocular disparities. Indeed, numerous investigations of this mechanism performed during recent decades discovered several cortical representations of neurons selective to absolute and relative binocular disparities in the primary visual area V1 (Barlow et al., 1967; Nikara et al., 1968; von der Heydt et al., 1978; Ferster, 1981; Gonzalez et al., 1993; Poggio, 1995; Prince et al., 2000), and in various extrastriate cortical areas (Poggio and Fischer, 1977; Maunsell and Van Essen, 1983; Andersen et al., 1985; DeAngelis and Newsome, 1999; Janssen et al., 2000; Thomas et al., 2002; Watanabe et al., 2002; Anzai et al., 2011; for the review see DeAngelis, 2000; Uka and DeAngelis, 2002; Tsutsui et al., 2005; Roe et al., 2007; Otero-Millan et al., 2014).

Another powerful mechanism for 3D reconstruction is the analysis of motion parallax (Toyama et al., 1986; Xiao et al., 1997; Cao and Schiller, 2003; Nadler et al., 2008). This mechanism dominates in animals with lateral eyes, as they do not have substantial overlap of visual fields. But it is also widely used in various situations by animals with frontal eyes. It was demonstrated that depth information extracted from analysis of motion and stereopsis could converge in activity of a single neuron (Bradley et al., 1995; Anzai et al., 2001; Pack et al., 2003; Ponce et al., 2008). Several studies explored other monocular cues of depth estimation (Tsutsui et al., 2002; Liu et al., 2004; Rosenberg and Angelaki, 2014).

However, in spite of this impressive increase in knowledge concerning the neural basis of 3D vision, there is a huge gap between the known properties of neurons involved in this function and ability of our depth perception. All investigated mechanisms of depth perception have a common limitation: they provide information about depth either relative to the location of the fixation plane or relative to the other objects in the visual scene, but not the absolute distance from an observer to an object. Thus, neurons selective to a particular disparity with one location of the fixating plane will be excited by objects at one distance, but after a shift of the fixating plain to another depth, the same neurons will respond to objects at another distance. At the same time our perception of depth is rather constant, and to some extent independent of the fixating planes. Therefore, some additional mechanism of depth constancy which would be able to extract behaviorally useful depth information from the actual retinal pictures using both retinal and extraretinal information, including past experience, seems to be necessary (Wallach and Zuckerman, 1963; Bishop, 1989; Collett et al., 1991; Predebon, 1993; Glennerster et al., 1998; Purves et al., 2015). Indeed, such ability was demonstrated in a behavioral study of monkeys (Li and Angelaki, 2005).

The simplest neuronal elements of depth constancy could modify the optimal disparity of the binocular cortical neurons, dependent on the distance from the observer to the fixation plane. The goal of such modification would be to keep constant absolute distance for neuronal activation, independent of the changes of the fixation planes. Such a mechanism has to obtain information regarding distance from the observer to the fixation plane. This distance can be derived, e.g., from the angle of vergence. At short distances it can also be estimated using information from the vertical disparities (Chowdhury et al., 2008). It was demonstrated that, in many cases, activity of disparity selective neurons was modulated, but they mostly showed modulations of the firing rate and not optimal disparity (Trotter et al., 1992; Genovesio and Ferraina, 2004). The possibility to extract egocentric distance using neurons with such properties was analyzed in the theoretical study of Pouget and Sejnowski (1994). Only in one study, and for only one neuron in area MST, a change of the vergence angle shifted the optimal disparity in the direction expected for depth constant neurons (Roy et al., 1992).

Although in all studies mentioned above visual stimulation was performed in well controlled conditions, these conditions differed significantly from natural animal vision. For such studies simple visual stimuli such as small bars or gratings were used, and the gaze was fixed and head movement restricted in order to provide reasonable visual stimulation of precisely mapped and often very small receptive fields. This was achieved either by paralyzing the eye muscles or by using, rather unusually for normal vision, long trained fixations of the gaze. While this approach allowed the opportunity to obtain essential information regarding the details of functioning of the particular mechanism in question, it restricted or completely eliminated the interactions between different mechanisms of depth estimation. Therefore, the information regarding depth coming from different sources could not be combined to create a consolidated perception of absolute depth. At the same time it was becoming obvious that responses to natural stimulation are not analogous to the ones observed for isolated and simplified stimuli, especially at later stages of visual processing (for the review see Reinagel, 2001; Felsen and Dan, 2005). One striking example of a difference in responses to natural versus standard random stimuli was offered by complex cells in the primary visual cortex. Their ability to detect oriented edges appeared to be higher for natural stimuli, which made complex cells a more sophisticated information integrator than was previously expected (Felsen et al., 2005). Natural conditions of viewing can be especially crucial for perceptual reconstruction of the 3D visual environment, since the importance of previous experience collected in phylo- and ontogenesis in natural conditions (Purves et al., 2015) has to be kept in mind.

It was reasonable to expect that neurons tuned to particular depth could exist somewhere in the visual system. Their activity would be independent of the distance to the fixation plane and the structure of visual stimuli. Some indications on the existence of such neurons were obtained in the studies which were conducted with behaving animals. To the best of our knowledge, the first description of the neurons which responded to visual stimuli and were tuned to a particular depth was given by Hyvarinen and Poranen (1974) in the parietal cortical areas of monkeys. They described neurons responding to stimuli presented exclusively within the near space, at the distance accessible by the hands of the animal. These neurons were not considered by the authors as elements of the visual depth constancy mechanism, but rather as elements important for organization of grasping behavior.

Following this work, neurons responding only to distant stimuli were discovered in the cat cortex, in the cortical area functionally equivalent to the monkey’s visual area V4 (Pigarev and Rodionova, 1991, 1998). These neurons being tested by stimuli of constant angular size responded only if the stimuli were presented at distances over 1.5 meters. Preferences to far located stimuli were observed both for the fixation point located at the same far distance or close to the animal face (although in the latter case the responses were decreased). Remarkably, this absolute depth preference was present even under monocular stimulation.

Since then neurons with visual responses modulated by distance were also found in the cortical area V4 of monkeys, where these neurons were considered as likely elements of a size constancy mechanism (Dobbins et al., 1998; Rosenbluth and Allman, 2002). Surprisingly, in this study, depth modulated neurons were found also in the primary visual cortex – area V1.

Cortical distribution of depth modulated visual neurons was investigated in our study in cats (Pigarev and Levichkina, 2011). Responses to visual stimuli of constant angular size were compared at near (30 cm) and far (3 m) distances for neurons in areas V1, V2, V4A and the frontal visual area (frontal eye field). Only in area V2 were neurons with depth modulated responses not found. In this study, cats were sitting on a trolley which could be located at different distances from the visual field. We noticed that sometimes neurons had the same level of background activity both for close and distant stimuli, but demonstrated strong excitation during motion at the intermediate distances. The simplest explanation of this observation was that these neurons had preference for a particular spatial frequency within the retinal receptive field, and they increased firing when this spatial frequency was achieved at the particular distance. This effect could also be connected with the drift of the retinal images during motion of a cat toward the visual scene. However, in the context of the previously mentioned observations, we did not exclude the possibility that some neurons could have absolute preference for stimuli located at the particular intermediate depth. The classical properties of visual receptive fields in the primary visual cortex (Hubel and Wiesel, 1962; Movshon et al., 1978) offered a simple experimental way to split these alternatives. Retinal drift could be excluded simply by greatly reducing the trolley speed. In turn, activity of every neuron could be studied during approach to two visual scenes with twice different spatial frequencies. If responses were defined by neuronal selectivity to spatial frequencies, maximal excitations in these two conditions should be observed at different distances. However, if neurons preferred absolute distance, they would be expected to have maximal excitation at the same location of the trolley, independent of the spatial frequency of the stimuli used.

We found that about 20% of neurons recorded in the area V1 in our experimental paradigm demonstrated preference to the absolute distances.

## Materials and Methods

Experiments were carried out with four adult cats using our modification of the technique for painless head fixation, first proposed by Noda et al. (1971). The technical procedure was described in detail earlier (Pigarev and Rodionova, 1998; Pigarev et al., 2009), and will be presented briefly below.

### General Design of the Experiments

**FIGURE 1:**
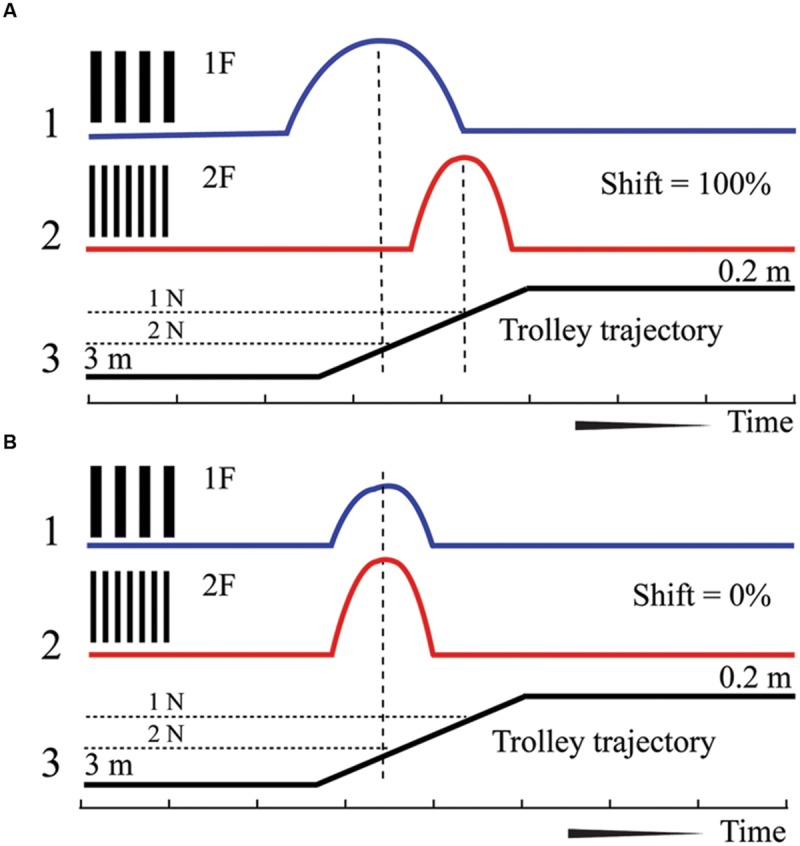
**Schematic drawing of responses expected for neurons with shifting maximums (A) and constant maximums (B).** 1 – Supposed spike density curve for the grating with low spatial frequency (1F), 2 – Supposed spike density curve for the grating with high spatial frequency (2F). 3 – Trajectory of the trolley movement from distant (left) to close (right) position. 1N and 2 N – two positions along the trajectory of trolley movement.

A cat was placed on a trolley. Neuronal responses from the primary visual cortex were recorded while the trolley with the cat sitting on it and facing the visual scene slowly approached the visual scene, which consisted of stationary sinusoidal gratings rear-projected over a large screen. Each neuron was tested with two gratings, with the spatial frequency of one grating being twice as high as that of the other. Assuming that a cell is tuned to a particular spatial frequency, the maximum of its response to the grating of higher spatial frequency should be shifted to a distance half way closer to the visual scene, in order to attain the same optimal size of the retinal projection on the retinal surface. However, if the neuron has selectivity to absolute depth, location of its maximum response should stay at the same distance irrespective of a change of the spatial frequency of the two gratings. This test and expected responses in these two types of neurons are schematically illustrated in **Figure 1**.

For exploration of the distant space, the animals were moved from the starting position at 3 m from the visual scene, and the presented gratings were of relatively large absolute size. Receptive fields of the studied neurons were located around 10° of eccentricities.

In experiments focused on the near space, the motion started from 103 cm from the visual scene. Receptive fields of the studied neurons were located in the central part of the visual field, within 3° of eccentricities. For this experiment we further reduced the speed of motion and absolute sizes of gratings.

### Shaping

The animals were familiarized with the laboratory space prior to the experiment. Before surgery they were placed in the lab for at least 1 week and were moving freely. They were also first fed while sitting on the standing trolley, and then while the trolley was moved at the same speeds used for the actual experiment. The shaping allowed us to eliminate anxiety and let the cats memorize the experimental environment.

### Surgical Procedure

Under deep general anesthesia (Pentobarbital sodium, 35 mg/kg, i.p.) the cat was placed in a stereotaxic device. The dorsal surface of the head was cleaned from soft tissue and small screws were inserted into the skull, about 15 mm to the right and left side from the midline. The screws were connected by thin wire, thus creating a frame around the central part of the skull. This wire frame was filled with self-hardening dental acrylic cement. The surface of the skull inside the frame was also covered by a thin layer of the same cement. The frame allowed access to the whole superior surface of the skull.

The experiments began a week after the operation. The animal was placed in a box, not limiting the body movements, and its head was restrained by fixation of the head frame in a special holder.

### Recording of the Neuronal Activity

Neuronal activity was recorded in the area V1 (cytoarchitectonic field 17) using plastic conic guiding tubes installed into a small hole (2 mm) in the skull and fixed to the plastic frame. Installation and construction of these tubes was described in detail in a previous article (Pigarev et al., 2009). Because there are no nociceptors in the skull and, therefore, no pain sensation, drilling of the hole for recording tubes can be done without any anesthesia. For the drilling we used slowly rotating devices and drill bits for metal. This allowed minimization of vibrations typical of fast dental drilling machines. During this procedure cats were fed in order to distract them. As a result, they did not pay attention to the drilling procedure and did not show signs of distress.

The first recording tube was located at the representation of the central part of the visual field, in the point with stereotaxic coordinates AP (- 4 mm), and laterality 1 – 2 mm. Positions of the receptive fields in this tube were estimated and, if necessary, the tube location was corrected according to the retinotopic map of the area V1 (Tusa et al., 1978). Positions of the receptive fields were estimated during voluntary fixation of the animal gaze on a piece of food.

Neuronal activity was recorded by varnish insulated tungsten microelectrodes (1–2 mΩ as measured at 1 kHz). In the first experiments a microelectrode was moved by hydraulic micro drive fixed to the head holder.

Later we used bipolar recording from two microelectrodes with their tips located at about 300–500 μm from each other. Microelectrodes were moved by mechanical micro drives fixed directly to the head frame. For spike recording we used amplifiers with differential head stages provided by NeuroBioLab Company. Local field potentials and spike activity, after filtering by incorporated band pass filters in the frequency range 0.3–2000 Hz, were stored on disk with high sampling rate (10 kHz) for further off-line analysis. Quality of the recording was monitored visually and aurally during the entire test session.

### Visual Stimulation

All neurons investigated were first checked for simple visual responses to bars (either real three dimensional or light projected onto a white screen), which were moved by hand while the cats were fixated on small pieces of food. After neurons with visual responses to these stimuli were found, we estimated their orientation preference. If neurons preferred vertical or tilted orientations we closed one eye of our cat to avoid possible influence of the “wall paper” illusion (McKee et al., 2007). In order to close one eye we arranged a small screen in front of this eye (about 2 cm from the sclera surface) to totally cover the main test screen. This covering screen was illuminated at the same level as our test screen and environment. In our previous study (Pigarev and Rodionova, 1998), it was noticed that depth dependent activity, although reduced, could be observed in monocular conditions too. When neurons preferred horizontal orientation, binocular visual stimulation was used. Detailed investigation of the receptive field properties was not done.

**FIGURE 2:**
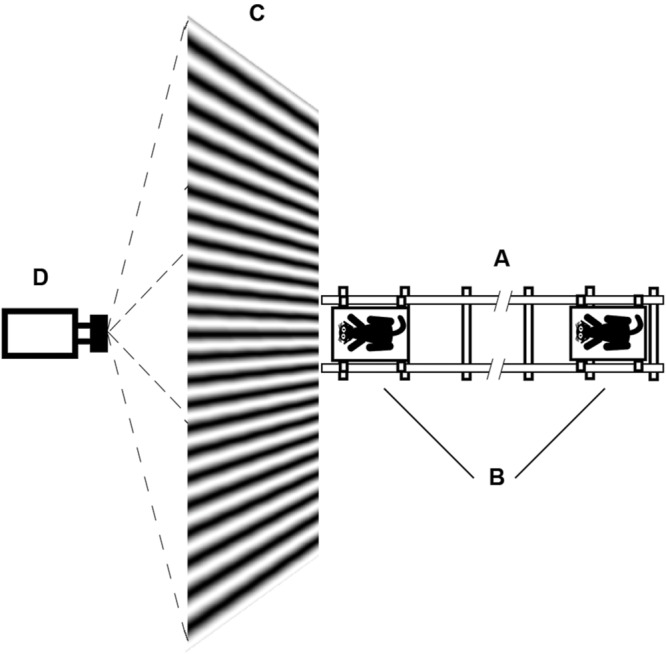
**Schematic drawing of the test procedure. (A)** Railway; **(B)** Trolley with animal; **(C)** Screen for rear projection; **(D)** Projector.

The animal holder and amplifiers were located on a trolley that was moved along a railway (**Figure 2**). Position of the trolley was recorded by string potentiometer, located along the railway, with its sliding contact fixed to the trolley.

Visual stimuli (sinusoidal gratings of various orientations and spatial frequencies) were rear-projected onto the special screen by video projector (**Figure 2**). This method of stimuli presentation let us move the animals close to the screen without casting shadows on the screen. The projector was fixed in such a way that the light beam was tilted about 30° to the horizontal plane. With this position of the projector, light flux in the direction of our cat was provided by uniform diffusion of light in the screen material, and the usual central spot with increased brightness for direct rear-projection was not visible from the animal’s point of view. The room was diffusely illuminated by lamps located high on the ceiling, and the mean illumination of the room was the same as the mean illumination of our test screen. Therefore, we believe we could assume that during trolley motion, not only illumination of the studied receptive field but also the general illumination of the entire retina stayed the same.

When the trolley was close to the visual scene, the screen covered nearly the whole visual field (160° in the horizontal direction). In the distant position (around 3 m), the screen covered only the central 60° of the visual field (30° in each hemi field). However, deviations of the gaze of more than 5° are very rare and short in time in cats. So, we were sure that both light flux and grating pattern within the area at least 20° around the investigated receptive field were identical during all test procedures. Other stationary elements of laboratory environment, which could be projected on the far retinal periphery, were also located approximately at the same distance as the main screen.

We intended to provide our animals with all possible clues for distance estimation to mimic natural conditions as fully as possible. To include memory cues we allowed cats to live and freely move in the laboratory space to familiarize them with the laboratory environment.

A test cycle was started with the trolley standing about 60 s distant from the visual scene. The trolley was then slowly moved toward the visual scene, and stayed there another 30 s. After that the trolley was moved quickly back to the starting position, spatial frequency of the grating was changed and the cycle repeated. Parameters of trolley motion were different in experiments to investigate the distant and near space, and will be given below. Neuronal activity was recorded in at least six repeated cycles with each grating and averaged. Further in the text we refer to each grating of each test as 1F if this grating had smaller spatial frequency, or 2F if it had twice higher spatial frequency. For a limited number of cells we managed to present three gratings with spatial frequencies of 1F, 2F, and 3F.

### Monitoring of Eye Movements and State of Vigilance

We used free gaze condition to study neuronal activity during natural voluntary observation of the uniform large visual scene with a particular spatial frequency at the particular distance from the animal. However, we monitored eye movements using an infrared oculometer, mainly in order to detect moments of eye closure due to reduced vigilance and developing sleep. In addition, we permanently recorded cortical EEG. It was previously shown (Pigarev et al., 1997) that sleep starts developing from associative and extrastriate cortical areas while area V1 still continues working in the way normal for wakefulness. Our electrodes for EEG recording were located over frontal and parietal cortices. So, first sleep spindles in the EEG indicated that our cats were becoming drowsy and may fall asleep soon also in area V1. If this occurred, we either stopped recordings, or tried to wake the cat. During data analysis we excluded trials if they were contaminated with sleep spindles.

### Gratings and Trolley Speeds Used in the Study

When the trolley moves toward the screen, projections of the stationary gratings drift in radial directions over the retinal surface, even if eyes are stable. The speed of such a drift primarily depends on the speed of trolley motion. This drift strongly accelerates when the trolley approaches the screen, and is also more pronounced for receptive fields of bigger eccentricity. The relationships of all these parameters are described by the simple formula which was obtained from the geometry of stimuli projections on the retinal surface for two close time moments

$$V_{1}\approx V_{2}\cdot \frac{E\cdot D_{1}}{D_{2}^{2}}$$

where V_1_ is the speed of the retinal drift expressed in mm/s, V_2_ is the speed of trolley motion in mm/s, E (eccentricity) is the distance of the receptive field from the center of gaze measured on the screen in mm, D_1_ is eye diameter (13 mm for our cats) and D_2_ is the distance from the eye to the screen in mm. Speed of the drift in degrees/s for the eye diameter of our cat can be easily calculated taking into account that the radius of the eye as an optical system is approximately equal to its diameter (13 mm in our case) which corresponds to 57° (radian) over the retinal surface. Therefore,

$$V_{1}(\text{degrees/s})\approx 4.35\cdot V_{1}\left(\text{mm/s}\right)$$

From classical studies of the visual receptive fields in cat area V1 (Hubel and Wiesel, 1962; Movshon et al., 1978) we knew that distribution of the optimal spatial frequencies in the central part of area V1 in cats varies from 0.25 to 2 cycles/degree with maximums around 0.7 cycles/degree, and speed preference vary from 1 to 16°/s with maximums around 4°/s. This let us estimate the range of stimulation parameters which could be used in this study. It became clear that in order to eliminate the impact of retinal drift at short distances, the trolley speed should be very low. On the other hand, with such a low speed of trolley motion, the duration of one trial became enormously long. It was obvious that with constant speed of trolley movement it would not be realistic to combine investigation of the distant and near spaces in a single experiment. Therefore, we have divided this study in two parts.

In the study of the distant space the speed of the trolley was 1 cm/s and locations of the receptive fields were within the range of up to 10° from the center of gaze; the distance from the eyes to the screen ranged from 2.8 to 0.2 m. However, according to our calculations, with this speed of trolley movement the retinal drifts of stationary stimuli related to motion could influence neuronal firing from 0.6 m. Indeed, in many neurons we observed an increase in firing rate at short distances. Absolute sizes of grating periods used in this block of studies were 38 mm for 1F and 19 mm for 2F. These values corresponded to spatial frequencies 1.2 and 2.4 cycles per degree at the distance 2.8 m, and 0.09 and 0.18 cycles per degree at 0.2 m. One trial lasted about 4.3 min, with about 1 min intervals between trials. This resting interval was needed to reward a cat with a small piece of food to keep it awake and alert during the experiment, as cats sitting with their head fixed have a natural tendency to fall asleep. The first part of the experiments was performed with two cats (Cats 1 and 2).

To study cell responses in the near space, we reduced the speed of trolley motion to 1.7 mm/s and reduced the length of trajectory. Now the starting position was located at 1.03 m from the screen, and the trolley moved to within 0.11 m of the screen. Even so the duration of one trial was about 9 min. For this part of our study we took receptive fields located not more than 3° from the center of gaze. Absolute sizes of gratings were also reduced. Their periods were 12 mm for 1F, and 6 mm for 2F, which corresponded to spatial frequencies 1.5 and 3 cycles per degree at far distance and 0.15 and 0.3 cycles per degree at short distance. This part of the experiments was performed with two cats (Cats 3 and 4).

### Data Analysis

Data was band pass filtered from 0.3 to 200 Hz (plus 200 Hz transition area) in order to get low frequency components of the recorded activity. Then from the original data we subtracted this low frequency component in order to get the channel with spike activity. Following this, we performed analysis of the shapes of recorded neurons using a spike-sorting algorithm offered by the Spike 2 program (Cambridge electronic design, UK). This procedure, which included principal component analysis of the spike shapes, resulted in several channels of single unit activity.

**FIGURE 3:**
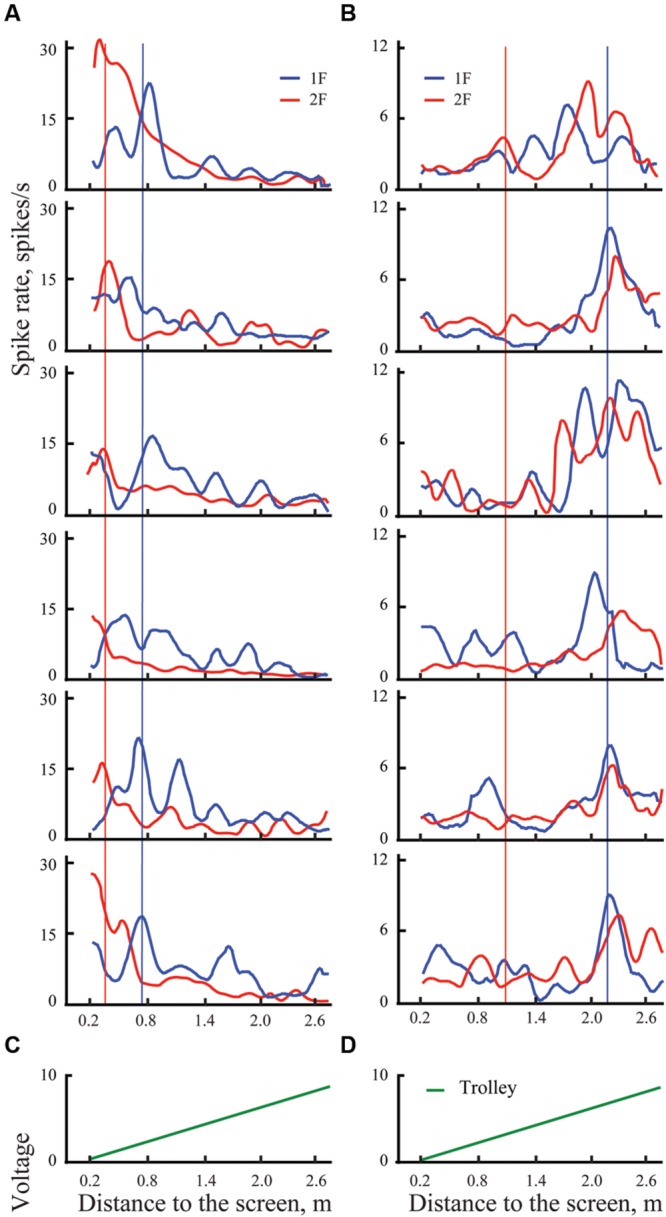
**Examples of spike rates for individual trials for presentation of two gratings in distant space, one with spatial frequency 1F (blue lines) and another with spatial frequency 2F (red lines) for two cells, one with shifting maximum (A), and another with constant maximum (B).** Left panel shows individual trials of the cell with shifting maximum for six pairs of trials (six trials with 1F and six with 2F). Vertical blue lines indicate the position of the mean maximum of cell activity for all trials with 1F (averaged across trials); vertical red lines represent the expected position of 100% shift (half distance between 1F maximum and the screen). Right panel shows individual trials of a cell with constant maximum, arranged in the same way. **(C,D)** Records of the trolley movement (green lines).

**FIGURE 4:**
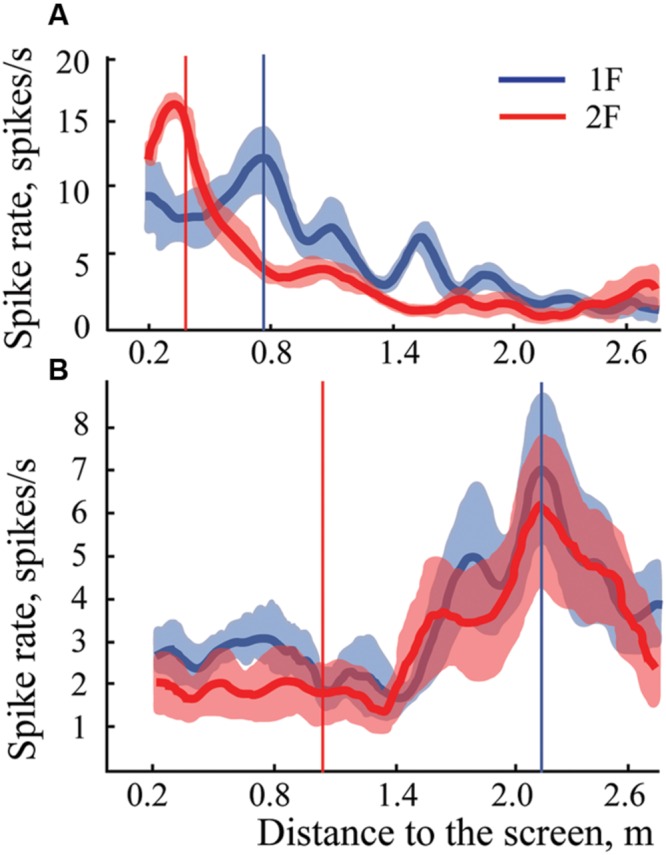
**Examples of mean spike rates (±SEM) for presentation of two gratings in distant space, one with spatial frequency 1F (blue lines) and another with spatial frequency 2F (red lines) for two cells, one with shifting maximum (A), and another with constant maximum (B).** Vertical blue lines indicate the position of the maximum on the blue curve (frequency 1F); vertical red lines show the position of the expected shift of the maximum with grating frequency 2F. Individual trials for these two neurons are shown in **Figure 3**.

For each neuron we transformed the sequence of single spikes into a spike density function using a sliding window of 20 s with 0.01 s step. For the studies of the distant space, this window corresponded to 18 cm of the trajectory. For the investigations of the near space the window corresponded to 3.4 cm. Trials obtained with a grating of a particular spatial frequency (1F or 2F) were averaged to get the mean spike rate curves of neuronal activity along the trajectory (**Figures 3, 4, 6**, and **9**). It is important to note that even if the neurons had very strong responses to visual stimuli presented inside their receptive fields, they often had very low ongoing firing, and sometimes did not have such activity at all. These nearly silent and often rather long intervals, when stimuli were outside the receptive fields, were averaged together with strong responses, as our smoothing window had to be long enough to pick up the distance-related changes of activity but not the accidental short peaks of the cell firing that can be present in the free gaze condition. As a result, mean firing rate may appear lower than expected for the area. This means that in the presented figures, one should estimate not the absolute values of the mean firing rate, but the relative values of firing rates at optimal and suboptimal positions. The maximum of such averaged activity along the track was used as an indicator of the optimal conditions for the cell response to a particular grating, in conjunction with the distance to the screen.

Statistical significance of every observed maximum of activity was tested using the bootstrapping method applied to each time window of every trial; the method is provided by the Matlab-based Chronux toolbox. Chronux toolbox is open-source software available from http://chronux.org/ (“Observed Brain Dynamics,” Partha Mitra and Hemant Bokil, Oxford University Press, New York, NY, USA, 2008). Using this method, the response maximum was considered significant if it exceeded 95% confidence level (bootstrap error bars) for the mean response rate. From 383 neurons recorded in four animals, 123 neurons demonstrated significant maximums for both gratings at particular parts of the trajectory.

We then calculated the shifts of the response maximum locations in the cases of two gratings with spatial frequencies 1F and 2F. This analysis was performed only if maximums for each of two presented gratings were significant. As the individual trial outcomes are independent, the probability of obtaining two significant maximums in one neuron by chance is a product of probabilities of each of them (0.05 × 0.05 = 0.0025). In our experiments, from 383 neurons recorded in four animals, 123 neurons demonstrated significant maximums for both gratings at particular parts of the trajectory, which clearly was above chance level.

The shift of the maximums between two gratings was given in percents and was calculated as:

$$\text{Shift}=\frac{\text{M1}-\text{M2}}{\text{M2}}\cdot 100\%$$

where M1 is the distance from the screen to the position of the maximum for the grating with the lower spatial frequency (1F), and M2 is the distance from the screen to the position of the maximum for the grating with the higher spatial frequency (2F).

We expected to find two categories of cells and two typical shift values respectively: cells tuned to particular spatial frequency should have shifts close to 100% (**Figure 1A**, shifting maximums), whereas cells tuned to absolute depth should demonstrate shifts close to 0% (**Figure 1B**, constant maximums). In our experiments we found a substantial number of both cell types.

At the next step of analysis, statistical assessment of the significance of the shift values was performed for every cell with both maximums found significant at the previous step. To do this for every cell, we created a distribution of all shifts by taking all individual trial values of the position of maximum for each of the gratings. As the trial values can be considered independent from each other due to the long duration of each trial, we could calculate all possible shift values of the maximums between the gratings (e.g., for six trials per grating the number of all possible shifts would be 36). We considered cells with shift values above 70% of the possible shift as having shifting maximums. To test significance of the shift, we performed a Sign test with H0 that the M1 – M2 distance distribution has a median not less than zero plus 70% of the shift. Cells with M1 – M2 shift values below 30% were designated as having constant maximums. We tested significance of constant maximums using a Sign test with H0 which states that the M1 – M2 distance distribution has a median not greater than zero plus 30% of the expected shift.

The reason for the allocation of a 30% range from the expected value comes from the empirical data. We used the group of classical frequency-selective cells that are always present in V1 to estimate the allocation range. To define this group, we crudely divided the distribution of shifts obtained for all cells into two halves, and the group with shifts above 50% was considered likely to consist of the classical frequency-selective cells. The standard deviation for the mean shift in this group was about 30% for all 4 cats (due to non-random distribution of maximums, see Results). We considered this range as the level of intrinsic noise resulting from the free gazing condition. We decided to treat possible new type of cells (cells with constant maximums) the same way as the well known frequency-selective type of cells, and used the same range for constant maximums.

All data reported here therefore had to satisfy two statistical criteria: for the individual significance of both response rate maximums (for two gratings) and for the significance of the shift classification.

### Animal Care

Surgery and day-to-day treatment of the animals were carried out in accordance with Ethical Principles for the maintenance and use of animals in neuroscience research (1987), NIH guidelines for the care and use of animals, Declaration of Helsinki on Ethical Principles for Medical Research Involving Human Subjects. Current Russian laws do not bind scientific Institutes to have special Ethic committees. However, evaluation of Ethics is conducted by the Institutional Scientific Council in the course of a decision concerning inclusion of a study in the plan for financial support. In grant distributing Foundations, Ethic evaluation also should be carried out by a Council of reviewers as part of a decision concerning financial support of a study. These Councils in general are guided by recommendations of the abovementioned documents.

## Results

### Part 1: Neuronal Activity in Distant Space

We recorded neuronal activity from the cat striate visual cortex (area V1, cytoarchitectonic field 17) during slow approach of the animal toward the visual stimuli. Every neuron was tested with at least two gratings (for details see Materials and Methods), and every grating was presented 6–10 times. From 85 cells studied in the first cat (Cat 1), 38 cells demonstrated significant increases of their firing rates (significant maximums) at a particular position along the trajectory. Forty seven cells did not have any changes of their activity dependent on the distance/spatial frequency. From 81 neurons studied in Cat 2, 20 neurons had a significant increase of firing rate dependent on distance/spatial frequency, while 61 cells did not have significant changes in mean firing rate.

**Figure 3** gives an example of 12 individual trials (six trials for each grating) recorded in experiments with two neurons (shown in two columns **Figures 3A,B**). Blue curves show spike density curves recorded during presentation of the grating of the lower spatial frequency (1F), and red curves represent activity during presentation of the grating with double spatial frequency (2F). These gratings were always presented in alternating sequence. The trials are shown as pairs in each panel to provide a picture of the response shifts from 1F to 2F gratings, as well as making the figure more compact. For the statistical analysis they all were considered independent.

Both these neurons had strong excitations at particular parts of the trajectory, which were recognized as significant after statistical tests. Blue vertical lines indicate positions of the maximums obtained for these neurons after averaging of all trials with grating 1F presentation (shown in **Figure 4**). Red vertical lines indicate the positions to which shift of the maximums was expected after changing the grating to double spatial frequency (2F), in accordance with geometrical considerations.

Response maximums of the neuron shown in column A, which were obtained during presentation of the 2F grating (red curve), fluctuated around the red vertical line, thus indicating that activity of this neuron most likely was defined by selectivity of the receptive field to the particular spatial frequency of the stimulus. This was confirmed by the statistical test. Therefore, this neuron was classified as having shifting maximum. The response maximums of a cell with constant maximum are shown in column B.

In Cat 1, 16 out of 38 cells with significant maximums for both gratings were characterized as having significant constant maximums and 16 as having significant shifting (and the remaining six cells did not pass shift significance criteria). In Cat 2, constant maximums were observed in 8 out of 20 cells and shifting maximums in nine cells (three did not pass the shift significance criteria). These results are summarized in **Table 1**.

**Table 1:** The distribution of the different cell types investigated in four cats.

	N cells studied	N cells with significant maximums	N cells with constant maximums	N cells with shifting maximums
Cat 1	85	38	16	16
Cat 2	81	20	8	9
Cat 3	94	44	19	21
Cat 4	123	21	18	2
Total	383	123	61	48

**FIGURE 5:**
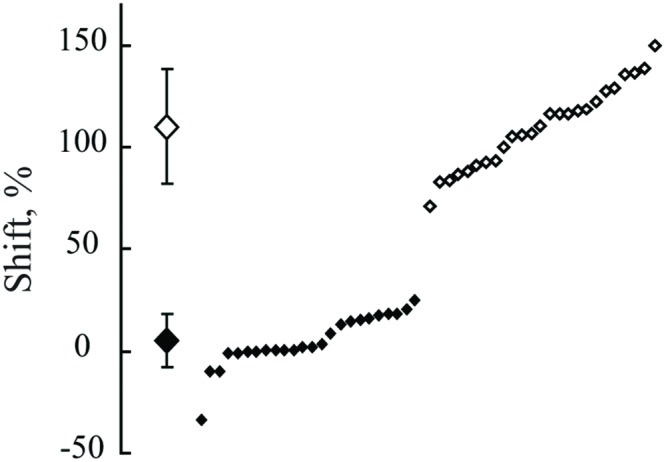
**The distribution of shifts observed in positions of maximums after spatial frequency of the presented gratings was changed from 1F to 2F.** Data for all neurons with significant maximums in both Cats 1 and 2 are pooled together. At the left side, along the vertical axis, mean values of the obtained shifts for two groups of cells and their standard deviations are shown. Individual shifts for each of the cells (sorted in ascending order) are presented at the right part of the figure. Filled rhombuses correspond to cells tuned to absolute distance and empty ones represent cells tuned to spatial frequency.

**Figure 5** demonstrates the distribution of shifts in spatial locations of the maximums in response to changes of the spatial frequency of the test gratings for all the cells having significant maximums in both cats. The obtained distribution is clearly subdivided into two clusters. Shifts of the first cluster are concentrated around 0% and, in the second cluster, around 100%. We did not find any shifts in the range from 26 to 71%. Data collected from either of the two animals demonstrate good separation of the shift values into clusters of constant and shifting maximums.

A Lilliefors test applied to the whole set of shift values showed that shifts are not distributed normally (*p* < 0.001), being pooled together. On the other hand, individual Lilliefors tests for these two clusters (below and above 50% shift value) revealed that each of them had normal distribution (*p* > 0.05). Thus, we concluded that the obtained shift values belonged to two different groups of cells. For each group the measured values were normally distributed. Cells with shifts less than 30% were categorized as neurons with constant maximums (mean shift = 6.5%; *SD* = 13.3), tuned to absolute distance to the screen. Cells with shifts more than 70% (mean shift = 113%, *SD* = 30.3) were categorized as neurons with shifting maximums, tuned to spatial frequency.

**FIGURE 6:**
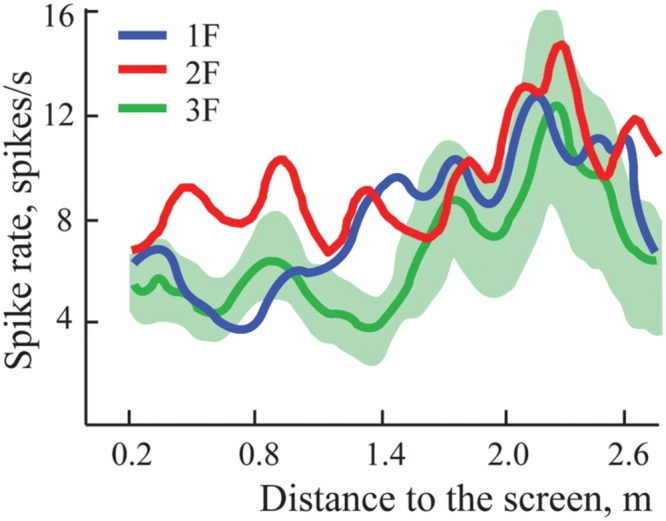
**Constant maximums observed on averaged spike rate curves of one neuron during approach to three different gratings with spatial frequencies 1F, 2F, and 3F.** Standard errors of the mean are shown only around the curve representing activity during approach to grating 3F, where values were greatest.

In some experiments we managed to demonstrate three gratings with spatial frequencies 1F, 2F, and 3F. For four neurons, maximums for all three gratings passed the test for significance and were classified as constant maximums. **Figure 6** shows results obtained in experiments with one such neuron. It is seen that all three curves have clear activation around 2.2 m independent of the gratings used.

**FIGURE 7:**
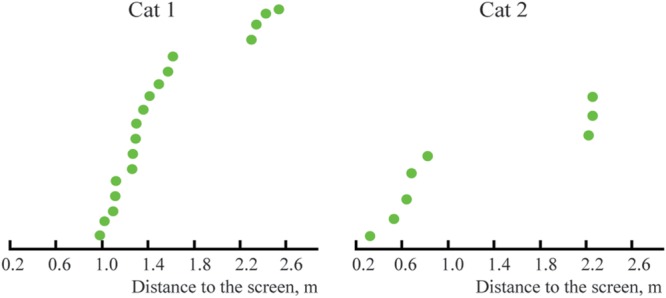
Distribution of distances from the screen to locations of constant maximums found in Cats 1 and 2 (sorted in ascending order).

**Figure 7** represents the distribution of distances from the screen of the locations of the constant maximums for the cells recorded in Cats 1 and 2. We found that positions of these maximums were not distributed uniformly along the trajectory, but had a tendency to group in clusters. For every cat, locations of all recorded maximums did not fit a single normal distribution (Lilliefors test, *p* < 0.05).

### Part 2: Neuronal Activity in Near Space

In this part of our study, experiments were performed with neurons having receptive fields within 3° from the center of gaze. We reduced the absolute sizes of the gratings (see Materials and Method), trolley distance range (1.03 – 0.11 m), and speed of the trolley motion (1.7 mm/s).

In these conditions, in Cat 3 we recorded activity of 94 cells, and 44 of them had significant maximums for two or three gratings. Nineteen cells were classified as having significant constant maximums and 25 as having significant shifting maximums. Eight of 19 cells with constant maximums were tested with three gratings, and had significant constant maximums for gratings with spatial frequencies 1F, 2F, and 3F.

Four out of 25 cells with putative shifting maximums were excluded from the analysis as outliers, as their maximums for higher spatial frequencies were observed closer than one half of the distance from the starting position. Their response rates for gratings with lower spatial frequencies monotonically decreased immediately after the beginning of motion, and precise locations of the maximums could not be accurately measured. The expected positions of maximal activity for these cells should be located at distances exceeding the maximal distance of our trolley trajectory (103 cm). Therefore we decided to report 21 cells with shifting maximums for Cat 3.

In Cat 4 we recorded activity of 123 cells. Twenty one cells had significant maximums for both gratings, 18 of them classified as having constant maximums, and 2 as having shifting maximums, 1 did not pass the criteria for significance of the shift. These results are summarized in **Table 1**.

**FIGURE 8:**
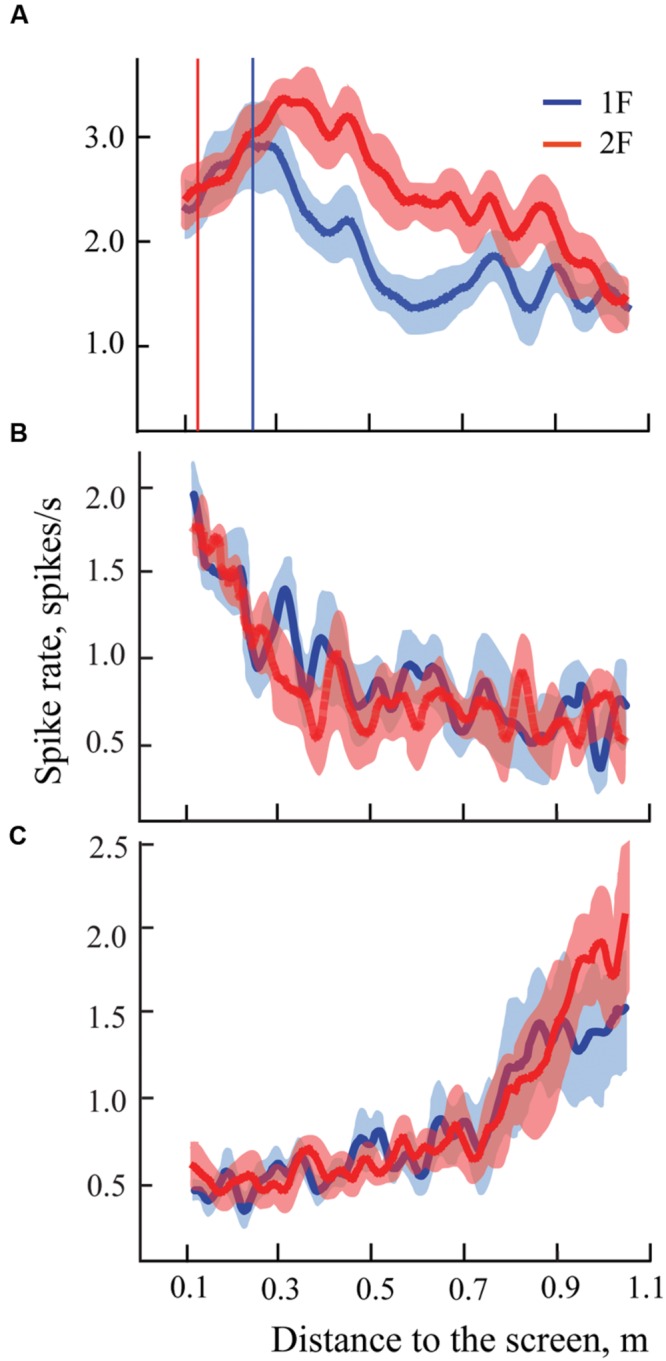
**Examples of mean spike rates (±SEM) for presentation of two gratings with spatial frequency 1F (blue lines) and 2F (red lines) for three cells recorded in near space.** Vertical blue line indicates position of the maximum on the blue curve (frequency 1F); vertical red line indicates position of the expected shift of the maximum for grating frequency 2F. Other details are in the text.

**FIGURE 9:**
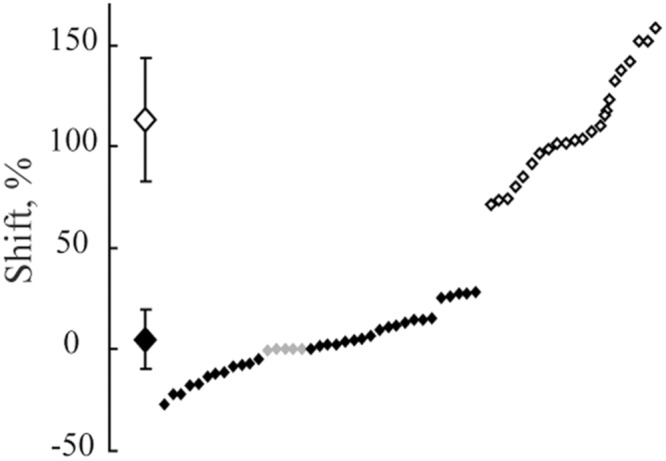
**The distribution of shifts observed in positions of maximums after spatial frequency of the presented gratings was changed from 1F to 2F.** Data for all neurons with significant maximums and significant shift values from Cats 3 and 4 are pooled together. At the left side, along the vertical axis, mean values of the obtained shifts for two groups of cells and their standard deviations are shown. Individual shifts for each of the cells (sorted in ascending order) are presented at the right part of the figure. Filled rhombuses correspond to cells tuned to absolute distance and empty ones represent cells tuned to spatial frequency. Gray rhombuses represent values for cells with activity increasing monotonously toward the closest position to the screen.

**FIGURE 10:**
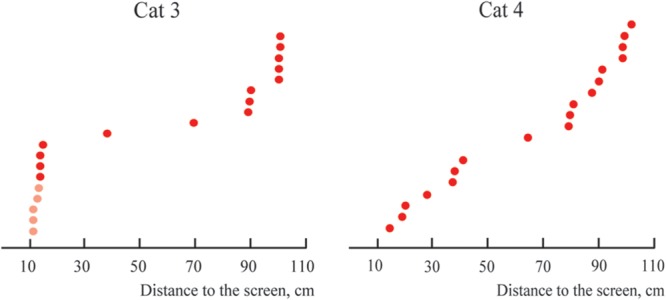
**Distributions of distances from the screen to positions of constant maximums found in the near space for Cats 3 and 4 (sorted in ascending order).** Distances for cells with activity increasing monotonously toward the closest position to the screen are shown in pink.

Three examples of spike density curves recorded in near space are shown in **Figure 8**. Activity of the typical cell with constant maximum is shown in fragment **Figure 8A**. In fragments **Figures 8B,C**, two neurons with monotonic types of activity changes along the trajectory are shown. The first of these neurons (**Figure 8B**) demonstrates increasing firing rate up to the end of the trajectory. Five cells with this type of response were recorded in this part of our study. These neurons were classified as neurons with constant maximums at the minimal distance (11 cm). This particular cell type, potentially important for distance estimation, will be discussed in full detail later (see Discussion). These five cells with monotonic change of activity are shown by faded colors in all the figures that represent them (**Figures 9, 10**, and **12**).

Neurons with activity rising toward the furthest distance (**Figure 8C**) most likely also had constant maximums, but they were located outside our test range (beyond 103 cm). However, we could not exclude the possibility that their maximums were shifting. Because of this, these neurons were not included in the analysis.

**Figure 9** demonstrates distribution of shifts in spatial locations of the maximums in response to changes of the spatial frequency of the test gratings for all the cells having significant maximums in Cats 3 and 4. The obtained distribution again is clearly subdivided into two clusters (compare to **Figure 5**).

The Lilliefors test showed that shifts which were observed in all cells with significant maximums in Cats 3 and 4 were not distributed normally (*p* < 0.001), similar to the results for the distant space. However, this test performed individually for each cluster revealed that they did not differ from normal distribution (*p* > 0.05).

**Figure 10** represents the distribution of distances from the screen to the locations of the constant maximums for the cells recorded in Cats 3 and 4. Again, as for Cats 1 and 2, positions of these maximums are not distributed uniformly along the trajectory, but have a tendency to group in clusters. For every cat, locations of all recorded maximums did not fit a single normal distribution (Lilliefors test, *p* < 0.05).

## Discussion

### Neurons with Absolute Selectivity to Depth – Imaginary or Real?

The main knowledge concerning functional organization of the visual system, and particularly of visual analysis of the 3D environment, is based on results obtained in well controlled conditions of receptive field stimulation. However, these conditions were very far from the natural functioning of the visual system, and the stimuli used were also rather artificial. In natural conditions, it is possible that neurons with other properties could be found, and that neurons with well known properties might behave differently. Theoretical aspects of this problem were reviewed, for example, in Reinagel (2001), Felsen and Dan (2005), Hollingworth (2006), Geisler (2008). In order to test this theoretical assumption several experimental studies were conducted during the last two decades. In one approach, the impact of natural visual stimuli in comparison to the traditional simple stimuli was investigated while the eyes were stabilized (Vinje and Gallant, 2002; Felsen et al., 2005; Guo et al., 2005; Yao et al., 2007; Onat et al., 2011). In the other approach, main attention was directed at investigation of the effects of the natural process of viewing (MacEvoy et al., 2008; Rajkai et al., 2008). We found two studies where both natural stimuli and the natural process of viewing were combined (Maldonado et al., 2008; Sellers et al., 2015). In another study, features of stereopsis in the natural environment were investigated (Sprague et al., 2015). Results of all these studies showed the potential importance of natural conditions of stimulation for investigation of the visual system.

We also thought that in order to address an issue leading to absolute distance perception, it would be important to use the free gaze condition, and to present stimuli at behaviorally different distances from the animals. However, free eye movements, together with the long trials required, could produce a substantial level of unpredictable noise. As a result, the probability of finding neurons with preference to absolute depth looked rather unlikely. Even assuming that such neurons do exist, the shifts of the peaks of their tuning curves could be strongly influenced by the noise, and it would be difficult to decide to what extent the observed shifts still can be considered as belonging to units with constant responses to particular depth. Fortunately, the classical visual cells with selectivity to particular spatial frequencies of the stimuli helped us to solve this problem, and to validate our method of visual stimulation in free gaze conditions. If our method was reliable, after replacement of one grating with another with twice higher spatial frequency, the distance from the response maximum to the visual stimulus should be halved. We found a reasonably large population of cells with such responses, which demonstrated that our method of stimulation let us reproduce the well known properties obtained previously in perfectly controlled conditions of stimulation. It also allowed us to estimate the level of noise produced by the free gaze condition in our experiments. Any scatter around the geometrically predicted position of the maximums of these classical neurons could be caused by noise only, which presumably had comparable values for all cells recorded in these experiments. Thus, if the scatter of peak locations for neurons with constant maximums was equal to or less than the scatter observed for neurons with shifting maximums, one could conclude that neurons with constant maximums really do exist. This is what we observed in our experiments. Looking at standard deviations for mean values of shifts for these two groups of neurons (**Figures 5** and **9**), one can see that the scatter for neurons with constant maximums was even less than the scatter for neurons tuned to spatial frequencies.

**Figures 5** and **9** demonstrate that shift distributions, instead of having a single cluster of shifts randomly scattered around the 100% shift position, created two clear and non-overlapping clusters. This is probably the strongest argument in favor of the presence of neurons with preference to absolute depth in the primary visual cortex. One cluster was centered close to the predicted position of 100% shift, and the other was close to 0% shift. It is not likely that such a separation could occur by chance in all four cats studied.

Importantly, we also found that positions of tuning curve maximums obtained from neurons with constant maximums were not randomly distributed along the trajectory, but were again grouped in clusters (**Figures 7, 10**, and **12**). The clustering of the maximums along the depth scale is another indication of existence of some order, which fits well with the mechanism of depth coding discussed in the next section.

### Hypothetical Mechanism of Absolute Depth Coding in the Cerebral Cortex

Our results show the presence of neurons tuned to absolute depth in the cat primary visual cortex. Looking at the shapes of the spike density curves obtained for the neurons with constant maximums (**Figures 4B, 6**, and **8A,B**), one can see that this tuning is not sharp, and positions of maximums of these tuning curves are grouped in some clusters (**Figures 7, 10**, and **12**). As a result, the obtained profiles of the activity curves of these neurons can strongly overlap along the depth axis.

This lets us assume that the mechanism of depth coding used at the final stages of visual processing, when objects are represented in the absolute depth coordinates, may resemble the mechanism known for color coding. Perception of multiple color hues is based on estimation of relative excitations of a limited number of color selective cones (only three for most humans), which are also broadly tuned to wavelengths and have strongly overlapping tuning curves. When excitation of these cells changes in response to changing of the illumination level, their relative activities stay more or less the same, thus providing invariant color perception.

**FIGURE 11:**
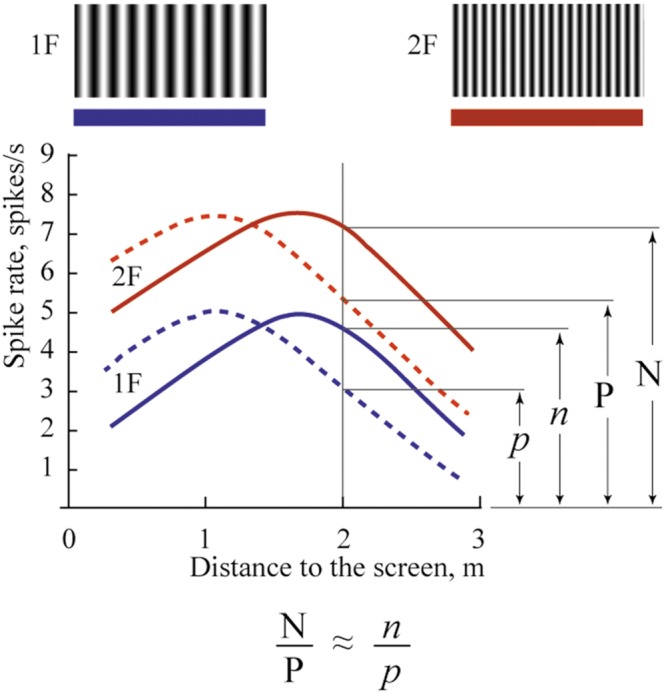
**Proposed mechanism of absolute depth coding.** Solid lines represent supposed responses to 1F and 2F gratings of one neuron with constant maximum; dashed lines represent responses of another such cell. Blue curves show activity during presentation of the grating with low spatial frequency (1F); red curves – grating with spatial frequency 2F. Explanations are in the text.

The mechanism of depth coding could be based on the same principle. This hypothetical mechanism is schematically shown in **Figure 11**. This figure shows spike density curves of two neurons with constant maximums at different distances from the eye to the screen. The tuning curve of the first cell is represented by the solid line and that of the second cell by the dashed line. These two neurons fire in response to two visual stimuli having different spatial frequencies (1F and 2F). Tuning curves in responses to the 1F grating are shown in blue and responses to the 2F grating in red.

In spite of the clear dependence of firing rate of these cells on distance, none of these cells alone can provide unambiguous information about distance to the visual stimulus. Indeed, analyzing the spike density curves in **Figure 11**, one can see that firing rates for distances symmetrically located around positions of their maximums are nearly the same. In addition, firing rates of these neurons in response to stimuli located at the same distances, but having different spatial frequencies, are different (compare red and blue curves for the same distance in **Figure 11**).

However, if we analyze not the individual firing rates of these neurons but their relative values, we should get robust measures of absolute distances, which will be reliable at a wide range of distances, and for various spatial frequencies of the visual stimuli. For example, in **Figure 11**, in response to stimulus with spatial frequency 2F located at 2 m distance, activation of the first neuron (solid line) is N spikes/s, and of the second neuron (dashed line), P spikes/s. If we change the spatial frequency of the stimulus from 2F to 1F at the same distance (2 m), firing rates of both neurons will be reduced to *n* and *p* spikes/s. However, the ratios N/P and *n*/*p* will be practically equal, and these relative values will provide constant information about the distance. This ratio can be invariant not only to spatial frequencies of the stimuli but also to the levels of illumination. Thus, in order to code information about the 3D visual scene, for every neuropixel (the neuronal cluster providing information about the minimal element of the 3D visual scene), we will need in addition to four units (which code three colors and brightness), a limited number of cells having non-sharp tuning to absolute depths and strong overlap of their tuning curves. This is what we found in this study.

### Eccentricity of Receptive Fields and Depth Coding

Our results obtained for the distant space show that positions of the recorded maximums were not randomly distributed along the distance axis, but in both cats formed at least two clusters with clearly separated locations (**Figure 7**). Using the above mentioned mechanism, neurons with activity described by curves with clusters in these locations can code the very wide range of distances, which exceeded 3 m. Distances greater than 3 m were not studied in our experiments. However, surprisingly, we found only one neuron with maximum activity at a relatively close distance (about 30 cm, Cat 2), although the trolley was moving to less than this distance (20 cm from the screen) in every trial. This was the opposite of what one might expect, namely that short distance, near the face of an animal, would have the most detailed representation.

**FIGURE 12:**
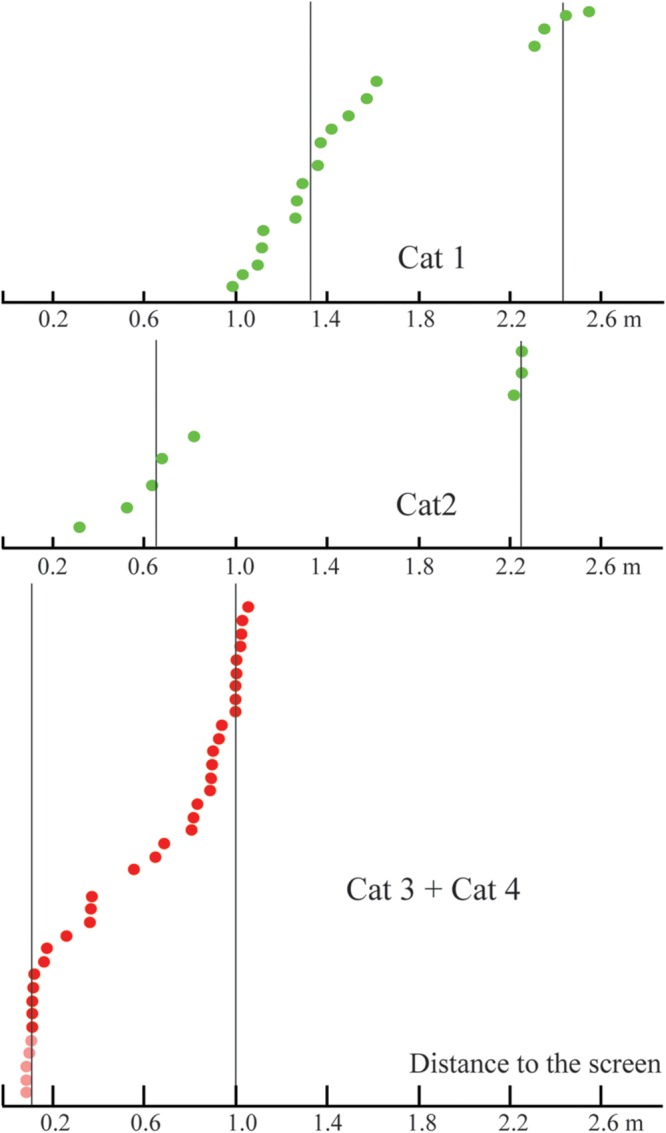
**Comparison of distances to the screen for constant maximums found in the distant space (Cats 1 and 2, green dots), and in the near space (Cats 3 and 4, red dots).** Vertical lines represent approximate locations of cluster centers. Distances for cells with activity increasing monotonously toward the closest position to the screen are shown in pink.

Numerous studies have shown that stereoscopic acuity and visual properties of neuronal receptive fields, even within a single cortical visual area, differ dependent on the eccentricity (Rawlings and Shipley, 1969; Joshua and Bishop, 1970; Orban et al., 1986; Battaglini et al., 1993; Rodionova et al., 2004; Palmer and Rosa, 2006; Xu et al., 2007; Yu et al., 2010). In our experiments focused on the distant space, all receptive fields tested were located around 10° from the center of gaze, and the gratings had relatively large absolute sizes. It is possible that fine estimation of depth at short distances could be performed, not for the whole visual field, but only for its central part. This fine depth coding could be applied to small objects located close to the animal. In order to test this hypothesis, we performed a second part of the study, where we focused on close distances and more centrally located receptive fields. **Figure 12** compares results obtained in both distant and near conditions.

### Depth Coding in Near Space and the Importance of Tuning Curve Flanks

In accordance with our expectation for the near space (Cats 3 and 4) we found many neurons with constant maximums at short distances. In addition, in these experiments we found a group of neurons which did not have the usual maximums, but which demonstrated a continuous increase of firing rate up to the minimal distance from the screen (**Figure 8B**). This group of neurons drew our attention, as several studies have presented theoretical considerations and experimental data in favor of the hypothesis that best stimuli discrimination should happen, not near the peak of neuronal tuning curves, but at the flanks of these tuning curves. Indeed, it is in the flanks, and not in the area of the maximal excitation, that small changes to the stimulus yield the largest changes in neuronal responses (Vogels and Orban, 1990; Schoups et al., 2001; Purushothaman and Bradley, 2005; Gu et al., 2007; Chowdhury and DeAngelis, 2008; Purushothaman et al., 2014).

**FIGURE 13:**
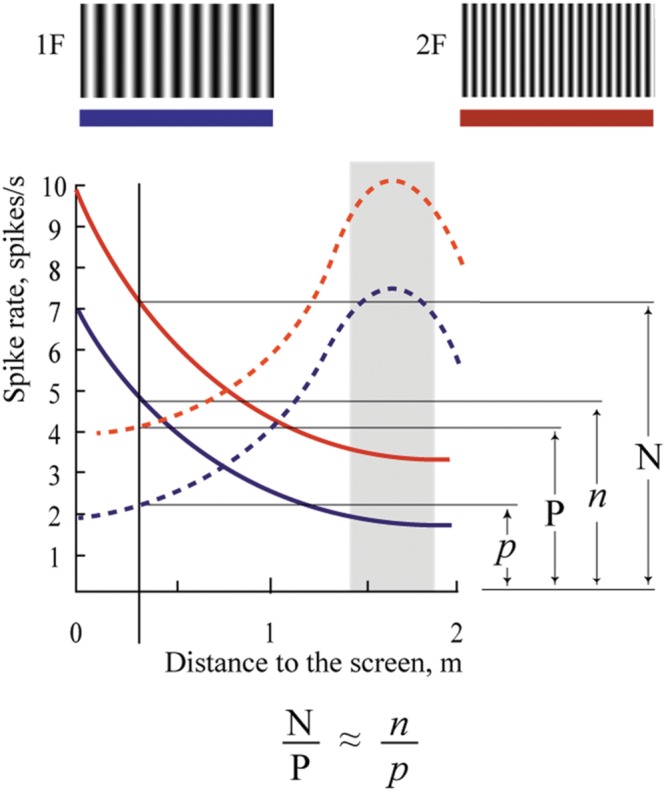
**Modification of the proposed depth coding mechanism for the near space.** Details are in the text.

The neuronal mechanism of depth coding which was proposed above would also be more efficient in the flank regions, where small changes of distance would lead to stronger changes in neuronal firing. Near the maximums, variations of the distance lead to small changes of neuronal firing rate. In **Figure 13**, the shadowed area highlights regions of spike density curves around the maximums for the neuron, shown by the dashed lines. The same shadowed area covers the tails of the curves for the neuron shown by the solid lines. It is unlikely that neuronal activity within the shadowed area can be used for efficient coding of depth information. On the other hand, to the left of the shadowed area all the curves have steep gradients, and can be used efficiently for coding of the distance using the suggested mechanism. It is obvious that in this area, minimal shifts of distance can lead to strong changes of the relative excitation values (situation indicated by the vertical line).

With the obtained data it is possible to speculate that in the central part of the visual field, information about fine depth is coded only for small objects located within short distances from the eyes, while toward the periphery, the ranges of the represented absolute sizes and distances to the objects are increasing.

### Possible Mechanisms of Absolute Depth Selectivity in Area V1

The observed pattern of neuronal activity cannot properly be explained by any single known mechanism of depth estimation. The neurons we recorded from were sensitive to binocularity of the inputs, as their responses in binocular conditions were stronger, but they preserved depth selectivity also in monocular conditions, which indicated that monocular mechanisms can also influence their responses. However, in monocular conditions in our paradigm, they could not use the efficient monocular mechanism of motion parallax. There is another mechanism which could be utilized in monocular conditions, namely accommodation. Our conditions of illumination and ranges of distance allowed use of this mechanism. However, we do not think accommodation actually is the main source of input in our case. In our experiments we used sinusoidal gratings, which appear defocused as a result of the absence of high frequency components of the gratings. It is known that high frequency components of images are the most important for efficient accommodation. So, it is not very likely that accommodative mechanisms alone could provide the absolute depth estimation observed in our experiments.

Most likely neurons with absolute depth selectivity described in our study used information from past experience, especially because our cats were well familiarized with the area and surrounding objects. In a similar way, a person barely notices changes in the perceived depth of their own room or office after closing one eye, and certainly will not lose awareness of the 3D structure of this familiar scene, or the ability to estimate distance to the well known objects in this room. So, it is possible to assume that some neurons in our brain also continue extracting depth-related information, even in monocular conditions.

In this case, these neurons most probably receive signals from other cortical or subcortical areas. The possibility of using such distance information feedback from other brain areas in area V1 was investigated in a recent theoretical study by Qian and Yazdanbakhsh (2015). This view can be supported by some observations made in our study. We noticed that neurons with constant maximums were often recorded in the first experiments, soon after the implantation of the new recording tube. In these experiments we usually stopped penetration when the first responding neurons were found. These neurons most likely were located in the superficial cortical layers. It is known that superficial layers receive projections from other cortical areas. In the deeper layers, neurons typically had classical simple and complex receptive fields, and their activities were dependent on the retinal spatial frequencies (neurons with shifting maximums).

In our opinion, the key approach for understanding of the neuronal organization of perception was offered by Purves et al. (2015). Neurons sensitive to absolute depth can be considered as elements realizing this “Wholly Empirical Paradigm” summarized in this article.

## Conclusion

Our study, which was performed in conditions of natural viewing with preserved eye movements, demonstrated the existence of neurons in the primary visual cortex whose activity was dependent on the absolute depth of the visual scene, and that this was independent of the spatial frequency of the visual stimuli used. Although in natural viewing conditions some uncontrolled variables might potentially cause a response elevation along the track, none of them could lead to the systematic effects observed. Therefore, we interpret our findings as an indication of absolute depth information coding in the primary visual cortex.

## Author Contributions

Both co-authors contributed equally to all steps of this study.

## Conflict of Interest Statement

The authors declare that the research was conducted in the absence of any commercial or financial relationships that could be construed as a potential conflict of interest.
